# Retraction: Role of alterations in protein kinase p38γ in the pathogenesis of the synaptic pathology in dementia with Lewy bodies and α-synuclein transgenic models

**DOI:** 10.3389/fnins.2025.1721408

**Published:** 2025-10-17

**Authors:** Frontiers Editorial Office

The journal retracts the 31 March 2020 article cited above.

Following publication, concerns were raised regarding the integrity of the images in the published figures. Areas of duplication were identified in supplementary figures 1C, 2B, 4A and 5C. The authors failed to provide a satisfactory explanation during the investigation, which was conducted in accordance with Frontiers' policies. As a result, the data and conclusions of the article have been deemed unreliable and the article has been retracted.

This retraction was approved by the Chief Executive Editor of Frontiers. The authors do not agree to this retraction.

